# Cross-sectional study of lung cancer patients as a potential high-risk factor for abdominal aortic aneurysm

**DOI:** 10.1371/journal.pone.0315898

**Published:** 2025-01-06

**Authors:** Hye Ran Gwon, A La Woo, Seung Hyun Yong, Young Mok Park, Song Yee Kim, Eun Young Kim, Ji Ye Jung, Young Ae Kang, Moo Suk Park, Du–Young Kang, Seong Yong Park, Sang Hoon Lee, Jun Seong Kwon

**Affiliations:** 1 Division of Pulmonology, National Cancer Center, Goyang, Gyeonggi, Korea; 2 Department of Internal Medicine, Division of Pulmonary and Critical Care Medicine, Severance Hospital, Yonsei University College of Medicine, Seoul, Republic of Korea; 3 Department of Thoracic and Cardiovascular Surgery, Kangbuk Samsung Hospital, Sungkyunkwan University College of Medicine, Seoul, Republic of Korea; 4 Department of Thoracic and Cardiovascular Surgery, Samsung Medical Center, Sungkyunkwan University School of Medicine, Seoul, Republic of Korea; 5 Department of Vascular Surgery, Charm Vascular Clinic, Seoul, Republic of Korea; Stanford University School of Medicine, UNITED STATES OF AMERICA

## Abstract

**Background:**

Abdominal aortic aneurysm (AAA) is more common in Non-small cell lung cancer (NSCLC) patients. Considering that ruptured AAA is potentially fatal, timely management of AAA would result in long-term survival benefits. We assess the prevalence and characteristics of AAA in resectable NSCLC patients who would benefit from AAA surveillance.

**Methods:**

1,019 resectable NSCLC patients in Severance and Kangbuk Samsung Hospitals were reviewed from January 2019 to November 2020. The control group comprised 2,899 cancer-free people who had a health check-up CT scan in Severance between January 2018 and December 2019.

**Results:**

Among resectable primary NSCLC patients, 39/1,019 (3.8%; odds ratio [OR], 19.19; 95% confidence interval [CI], 8.10–46.46) had AAA compared with 6/2,899 (0.2%) in the control (P<0.001). In multivariable regression analysis, male (OR, 13.24; 95% CI, 1.50–117.48; P = 0.020), aging (OR, 1.10; 95% CI, 1.04–1.15; P<0.001), current smoker (OR, 4.20; 95% CI, 1.20–14.62; P = 0.024), and coronary artery disease (OR, 3.13; 95% CI, 1.48–6.62; P = 0.003) were independent risk factors for AAA in NSCLC.

**Conclusion:**

The present study found that the incidence of AAA in resectable early-stage lung cancer patients was significantly higher than in the cancer-free control group. Therefore, we suggest that early-stage NSCLC patients, especially smokers older than 60 years, undergo regular AAA surveillance as part of their lung cancer monitoring.

## Introduction

An abdominal aortic aneurysm (AAA) is defined as the dilatation of the abdominal aorta to more than 3.0 cm in diameter [1,2]. Although the incidence and prevalence of AAA are generally low, the mortality of ruptured AAA is very high; The mortality rate of emergent surgery after rupture is more than 40%, and only 10–25% of them are likely to survive until discharge [1,2]. The growth rate and risk of rupture in AAA increase proportionally to the diameter, which increases over time. Therefore, patients with AAA on initial screening are recommended to undergo regular surveillance every 6 months to 3 years, depending on the aneurysm size [3,4]. This is because regular surveillance and timely intervention are important for survival in high-risk patients for AAA.

Lung cancer is one of the most common cancers worldwide and the death rate is higher than other cancers. Depending on the extent, the 5-year survival rate for localized-stage NSCLC is 57%, while the overall 5-year survival rate for all stages of lung cancer is only 5% [5–7]. The number of patients with early-stage lung cancer has increased owing to early diagnosis using low-dose computed tomography (LDCT) screening [8]. Consequently, the proportion of patients with resectable lung cancer has increased, and the prognosis of lung cancer has also improved [9].

Several risk factors of AAA, including smoking, male sex, older age, hypertension, dyslipidemia, coronary artery obstructive disease (CAOD), and chronic obstructive pulmonary disease (COPD), are also risk factors for lung cancer [10–12]. And, *Wiles B et al*. found that patients with lung cancer have a high prevalence of AAA. Therefore, we aimed to examine the prevalence of AAA and its characteristics in patients with early lung cancer who are eligible for resection.

Even though there is uncertainty in the assignment of cancer stage, a previous survival analysis of patients with lung cancer and AAA, with a median follow-up period of 6.13 years (interquartile range: 3.05–6.54), showed that AAA patients with lung cancer had a higher overall mortality risk than those in the matched non-AAA group [13]. It is well known that advanced-stage NSCLC havepoor 5-year survival rate (<5%, and <2% for stages IIIB and IV, respectively) [5–7]. Additionally, it is also known that the rupture of AAA is very fatal (about 59–83% of patients with ruptured AAA die before they can be admitted to a hospital), but the rupture rate of AAA with a diameter less than 5 cm is not common (0–6%/year depending on the diameter) [1,2,4]. Considering the aforementioned poor prognosis of advanced-stage NSCLC and uncommon rate of rupture in small AAA patients, it could be inferred thatthe life expectancy of patients with advanced-stage NSCLC would be determined primarily by the prognosis of lung cancer and not by the risk of potential AAA rupture. Because surveillance aims to reduce the potential risk of AAA rupture and it takes lots of effort for a lifetime, only early-stage NSCLC patients could benefit from AAA surveillance. Therefore, considering the cost-effectiveness of AAA surveillance, we included only resectable NSCLC patients with considerable life expectancy sufficient to benefit from AAA surveillance for our analysis.

## Materials and methods

### Patients

A total of 1,019 patients with primary non-small cell lung cancer (NSCLC) who underwent lung cancer resection were reviewed retrospectively. Since positron emission tomography-computed tomography (PET-CT) has been widely used recently for the clinical staging of lung cancer and they include non-contrast abdominal-pelvic computed tomography (APCT) imaging, it was possible to evaluate the presence of AAA in patients with lung cancer who are candidates for curative surgery.

And we obtained a cancer-free control group from people who underwent a health check-up CT scan in Severance between January 2018 and December 2019. Among the 10,811 individuals who underwent a health check-up at Severance Hospital between January 1, 2018, and December 31, 2019, we excluded 696 patients with underlying malignancies and 7,216 individuals without abdominal imaging (APCT or PET-CT). There were a total of 2,899 participants in the cancer-free control group. Their demographic and risk factor data (age, sex, smoking history, body mass index [BMI], and comorbidities such as hypertension, diabetes mellitus, dyslipidemia, COPD, CAOD, peripheral artery occlusive disease [PAOD], and chronic kidney disease [CKD]), and the imaging and results of their abdominal imaging were extracted from electronic medical records as much as we accessible. Current smokers were defined as those who still smoked at the time of lung cancer diagnosis, and former smokers were defined as those who stopped smoking before the diagnosis of lung cancer. Histologic types and stages of lung cancer were also analyzed.

The Institutional Review Board and Ethics Committee of Severance Hospital approved this study (IRB number: 4–2021–1430).

### Measurement of AAA

AAA is defined as a maximal axial aortic diameter >3 cm on computed tomography angiography (CTA). The maximum aneurysm diameter of the abdominal aorta derived from abdominal imaging was based on the outer wall-to-outer wall distance in the plane perpendicular to the path of the aorta [14]. We retrospectively reviewed and measured the diameter of the abdominal aorta according to the definition of maximum aneurysm diameter on abdominal imaging (APCT and PET-CT).

### Statistical analysis

Continuous variables are expressed as mean ± SD and categorical variables are presented as a percentage value (n/total). Comparisons of the prevalence of AAA between the lung cancer and cancer-free control groups were analyzed using the Pearson χ^2^ test. We analyzed the independent associations of multiple variables as a risk factor of AAA in patients with lung cancer using a univariable logistic regression model. Using factors that were significantly (*P*<0.05) related to the development of AAA in lung cancer in the univariable logistic regression analysis, we assessed the independent risk factors of AAA in NSCLC with multivariable logistic regression analysis. Adjusted odds ratios (ORs) with associated 95% confidence intervals (CIs) were calculated. An adjusted P-value<0.05 was considered statistically significant. Analysis was done using SPSS version 26 (SPSS, Chicago, IL, USA).

### Ethics approval and consent to participate

This study was performed in accordance with the Declaration of Helsinki. This human study was approved by The Institutional Review Board and Ethics Committee of Severance Hospital–approval (IRB number: 4–2021–1430). All parents, guardians or next of kin provided written informed consent for the minors to participate in this study.

## Results

### Baseline characteristics

**Fig 1:**
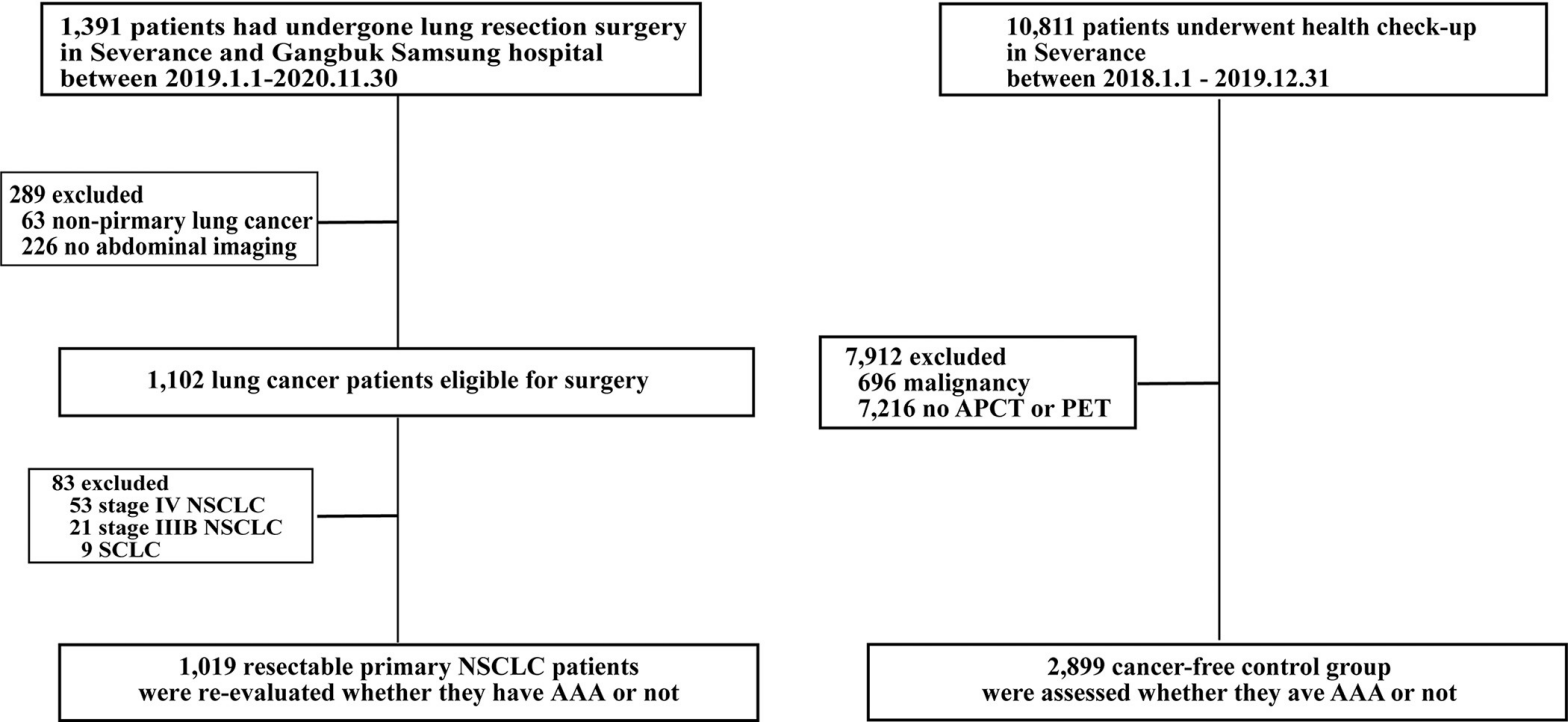
Flow chart. *NSCLC*, non-small cell lung cancer; *PET-CT*, positron emission tomography-computed tomography; *APCT*, abdominal-pelvic computed tomography.

1,391 patients underwent lung resection surgery at Severance Hospital and Kangbuk Samsung Hospital from January 2019 to November 2020. Among them, patients who did not have abdominal imaging (APCT or PET-CT), patients pathologically diagnosed as other than lung cancer, such as infection or metastatic lung nodules, patients who pathologically diagnosed as small cell lung cancer, and patients with unresectable (stage IIIB and IV) non-small cell lung cancer those who were undergo lung resection for palliative aim were excluded (Fig 1).

Among the 1019 patients with primary NSCLC who underwent lung resection, the mean age was 64.2 years (standard deviation 9.6 years) and 56.0% (571/1,019) were male. 33.6% (342/1,019) were former smokers and 15.8% (161/1019) were current smokers. The most common cancer stage was stage I (74.2%, 756/1,019). The most common cancer type was adenocarcinoma (81.8%, 834/1,019). Table 1 gives more information about other risk factors for AAA, histologic data, and cancer stage.

**Table 1:** Baseline characteristics.

		All patients	AAA	Non-AAA
		(N = 1,019)	(n = 39)	(n = 980)
Age, years		64.2±9.6	70.9±7.0	63.9±9.6
Sex	Male	571 (56.0)	38 (97.4)	533 (54.4)
	Female	448 (44.0)	1 (2.6)	447 (45.6)
Smoking history				
	Never	517 (50.7)	4 (10.3)	513 (52.3)
	Former	341 (33.5)	20 (51.3)	321 (32.8)
	Current	161 (15.8)	15 (38.5)	146 (14.9)
Smoking pack-years		19.6±25.0	36.8±20.1	18.3±25.0
HTN		479 (47.0)	29 (74.4)	450 (45.9)
DM		241 (23.7)	12 (30.8)	229 (23.4)
Dyslipidemia		236 (23.2)	14 (35.9)	222 (22.7)
COPD		133 (13.1)	19 (48.7)	114 (11.6)
CAOD		100 (9.8)	13 (33.3)	87 (8.9)
PAOD		11 (1.1)	1 (2.6)	10 (1.0)
CKD				
CKD stage 1		364 (35.7)	10 (25.6)	354 (36.1)
CKD stage 2		509 (50.0)	15 (38.5)	494 (50.4)
CKD stage 3		136 (13.3)	12 (30.8)	124 (12.7)
CKD stage 4		5 (0.5)	1 (2.6)	4 (0.4)
CKD stage 5		5 (0.5)	1 (2.6)	4 (0.4)
BMI, mean±SD		24.2±3.5	24.0±2.6	24.2±3.5
Obesity (BMI ≥30 kg/m^2^)		45 (4.4)	1 (2.6)	44 (4.5)
Cancer stage				
	Stage I	756 (74.2)	24 (61.5)	732 (74.7)
	Stage II	152 (14.9)	8 (20.5)	144 (14.7)
	Stage III	111 (10.9)	7 (17.9)	104 (10.6)
Cancer type				
	Adenocarcinoma	834 (81.8)	24 (61.5)	810 (82.7)
	Squamous carcinoma	152 (14.9)	13 (33.3)	139 (14.2)
	Other (non-small cell)	33 (3.2)	2 (5.1)	31 (3.2)

*AAA*, abdominal aortic aneurysm; *BMI*, body max index; *HTN*, hypertension; *DM*, diabetes mellitus; *COPD*, chronic obstructive pulmonary disease; *CAOD*, coronary artery obstructive disease; *CKD*, chronic kidney disease.

The prevalence of AAA in smokers and non-smokers was as follows; In total, 49.4% (503/1,019) of lung cancer patients had a positive smoking history. The mean number of smoking pack-years (PY) was 19.6 PY. A significantly higher proportion of smokers than non-smokers had AAA (7.0% [35/503] vs. 0.8% [4/516]; OR, 9.53; 95% CI, 3.38–27.14; P<0.001; Table 2).

**Table 2:** Rates of abdominal aortic aneurysm (AAA) in patients with a positive smoking history (current or former) vs. non-smokers.

	Smoker	Non-smoker	Total
AAA	35	4	39
No AAA	468	512	980
Prevalence, % (n/total)	**7.0% (35/503)**	0.8% (4/516)	1,019

Odds ratio [OR] for AAA, 9.57; 95%, CI 3.38–27.14, P<0.001.

Values are reported as n.

Pearson χ^2^ test was used to determine significance (P<0.05).

*AAA*, abdominal aortic aneurysm; *OR*, odds ratio; *CI*, confidence interval.

### AAA prevalence in lung cancer vs. cancer-free control group

The prevalence of AAA was 3.8% (39/1,019) in the lung cancer group and 0.2% (6/2,899) in the control group. Using the Pearson χ^2^ test, the lung cancer group had a significantly higher prevalence of AAA than the control group (OR, 19.19; 95% CI, 8.10–46.46; P<0.001; Table 3). In terms of AAA risk factors between the two groups, the mean age in the lung cancer group was significantly higher than that in the control group (64.2±9.6 vs. 54.4±10.3 years, P<0.001; Model 1 in Table 3). Therefore, we conducted additional analyses to adjust for confounding variables. We performed 1:1 propensity score matching for sex, age, and/or smoking status between the lung cancer group and the control group. Model 2 analyzes an age and sex-matched population. The prevalence of AAA was 3.8% (39/1,019) in the lung cancer group and 0.3% (3/1,019) in the control group. Using the Pearson χ^2^ test, the lung cancer group had a significantly higher prevalence of AAA than the control group (OR, 10.90; 95% CI, 3.88–30.62; P<0.001; Model 2 in Table 3). Model 3 examines a population matched for age, sex, and smoking status. The prevalence of AAA was 3.8% (39/1,019) in the lung cancer group and 0.4% (4/1,019) in the control group. Using the Pearson χ^2^ test, the lung cancer group had a significantly higher prevalence of AAA than the control group (OR, 13.48; 95% CI, 4.15–43.75; P<0.001; Model 3 in Table 3).

**Table 3:** Comparison of baseline characteristics and AAA prevalence between lung cancer and control groups.

	Model 1^†^		Model 2^‡^		Model 3^¶^	
	Lung cancer (n = 1,019)	Control (n = 2,899)	Lung cancer (n = 1,019)	Control (n = 1,019)	Lung cancer (n = 1,019)	Control (n = 1,019)
Male	56.0 (571)	57.5 (1,66)	56.0 (57)	50.9 (519)	56.0 (57)	51.3 (523)
Age, years	64.2±9.6	54.4±10.3	64.2±9.6	62.6±8.3	64.2±9.6	62.6±8.3
Smoker	49.3 (502)	47.3 (1,378)	49.3 (502)	43.8 (436)	49.3 (502)	41.7 (424)
Former	33.5 (341)	27.6 (800)	33.5 (341)	30.5 (311)	33.5 (341)	28.9 (294)
Current	15.8 (161)	19.9 (578)	15.8 (161)	12.3 (125)	15.8 (161)	12.8 (13)
AAA	3.8 (39)	0.2 (6)	3.8 (39)	0.3 (3)	3.8 (39)	0.4 (4)
OR* (95% CI)	19.19 (8.10–45.46), P<0.001		10.90 (3.88–30.62), P<0.001		13.48 (4.15–43.75), P<0.001	

*AAA*, abdominal aortic aneurysm; *OR*, odds ratio; *CI*, confidence interval.

† Model 1: unmatched.

‡ Model 2: age, sex matched propensity score matching.

¶ Model 3: age, sex, smoking status matched propensity score matching.

* OR (95% CI) for AAA in lung cancer group compared to control group.

Categorical variables are reported as a percentage (n).

Continuous variables (i.e., age) are reported as mean±SD.

Pearson χ^2^ test was used to determine significance (P<0.05).

### Risk factors for AAA development in patients with lung cancer

We evaluated clinical risk factors which might contribute to the development of AAA using univariable and multivariable logistic regression analysis among the 1,019 patients with primary NSCLC who underwent lung resection. In the univariable logistic regression analysis, male sex (OR, 31.87; 95% CI, 4.36–233.03, P = 0.001), increasing age (OR, 1.10; 95% CI, 1.05–1.15, P<0.001) and especially age >60 years (OR, 17.33; 95% CI, 2.37–126.83), smoking history (OR, 9.57; 95% CI, 3.38–27.14, P<0.001), smoking PY (OR, 1.02; 95% CI 1.01–1.03, P<0.001), HTN (OR, 3.15; 95% CI 1.65–7.09, P = 0.001), COPD (OR, 7.22; 95% CI, 3.74–13.93, P<0.001), CAOD (OR, 5.13; 95% CI, 2.55–10.35, P<0.001), CKD stage (P = 0.004), and lung cancer pathology (squamous lung cancer, OR, 3.16; 95% CI, 1.57–6.35, P = 0.005) were independent risk factors for AAA in lung cancer (Table 4). We analyzed the independent contributing factors to higher AAA prevalence in lung cancer using multivariable logistic regression of the significant (P<0.05) AAA risk factors identified in the univariable analysis. Because smoking history (yes/no), smoking amount (PY), and COPD are closely related to smoking, we only used smoking history (yes/no) in the multivariable logistic regression analysis. Male sex (OR, 13.238; 95% CI, 1.492–117.482; P = 0.020), increasing age (OR, 1.10; 95% CI, 1.04–1.15; P<0.001), smoking history (P = 0.021), and CAOD (OR, 3.13; 95% CI, 1.48–6.62; P = 0.003) were independent risk factors for AAA in lung cancer in the multivariable logistic regression model (Table 4).

**Table 4:** Univariable and multivariable logistic regression analysis of several risk factors for the presence of abdominal aortic aneurysm (AAA) in the lung cancer group.

	Univariable analysis				Multivariable analysis			
	OR	95% CI for OR		P	Adjusted OR	95% CI for adjusted OR		P
Sex, male	31.87	4.36	233.03	0.001	13.24	1.49	117.48	0.020
Age†	1.10	1.05	1.15	<0.001	1.10	1.04	1.15	<0.001
Age ≥60 yrs	17.33	2.37	126.83	0.005				
Obesity	0.56	0.08	4.17	0.571				
Smoking, PY	1.02	1.01	1.03	<0.001				
Smoking, y/n^‡^	9.57	3.38	27.14	<0.001				0.021
Former smoker	7.95	2.69	23.47	<0.001	1.72	0.52	5.76	
Current smoker	13.15	4.30	40.23	<0.001	4.20	1.20	14.62	
HTN	3.15	1.65	7.09	0.001				0.179
DM	1.46	0.73	2.92	0.289				
Dyslipidemia	1.91	0.98	3.74	0.058				
COPD	7.22	3.74	13.93	<0.001				
CAOD	5.13	2.55	10.35	<0.001	3.13	1.48	6.62	0.003
PAOD	2.55	0.32	20.45	0.377				
CKD stage^※^				0.004				0.384
stage2	1.08	0.48	2.42	0.862				
stage3	3.43	1.44	8.12	0.005				
stage4	8.85	0.91	86.49	0.061				
stage5	8.85	0.91	86.49	0.061				
Cancer pathology^€^				0.005				0.912
SCC	3.16	1.57	6.35	0.001				
others	2.177	0.49	9.63	0.305				

*CI*, confidence interval; *OR*, odds ratio; *PY*, pack years*; y/n*, yes/no; *HTN*, hypertension; *DM*, diabetes mellitus*; COPD*, chronic obstructive pulmonary disease; *CAOD*, coronary artery obstructive disease; *PAOD*, peripheral arterial occlusive disease; *SCC*, squamous cell carcinoma.

For continuous variables (age), OR represents the change for every 1-unit change in the independent variable (years). The dependent variable is the presence of AAA, and the independent variables are age, sex, smoking history, smoking amount (pack-years), and comorbidities related to well-known risk factors of AAA (chronic obstructive pulmonary disease, coronary artery obstructive disease, chronic kidney disease). Male sex, older age, smoking history, and CAOD were positive independent risk factors for AAA. Adjusted odds ratios are represented by the OR.

† Age at the time of surgery.

‡ Former & current smokers, compared to never-smokers.

^※^ Compared with CKD stage 1.

^€^ Compared with adenocarcinoma.

## Discussion

We observed that patients with early lung cancer had a significantly higher prevalence of AAA than that of cancer-free general population. Unlike previous studies, it is noteworthy that this study confirmed the same trends in Asian populations, especially in early-stage lung cancer patients who are candidates for surgical treatment. We evaluated adjusted OR for AAA prevalence by multivariable logistic regression analysis among the 1,019 resectable primary NSCLC. Old age (especially >60 years), male sex, smoking history, and presence of the CAOD were independent risk factors for AAA development in patients with lung cancer. Given that long-term survival rates are lower in lung cancer patients with AAA than in those without AAA [13], the significant association between AAA and early-stage lung cancer indicates that optimized screening for AAA in patients with lung cancer could potentially improve long-term survival rates after cancer cure.

The baseline characteristics of AAA patients in each group are described in the S1 Table.

Multivariable logistic regression analysis revealed that the prevalence of AAA was 10-fold higher in the lung cancer group than in the cancer-free control group, even after adjusting for age, sex, smoking history, and other AAA risk factors (HTN, DM, and CAOD) (Table 4). This result indicating that lung cancer is also an independent risk factor for AAA development.

The key pathologic characteristics of AAA include vascular inflammation, oxidative stress, destruction of the aortic extracellular matrix (ECM), and thinning of the aortic wall with the loss of vascular smooth muscle cells [15]. The risk factors for AAA in lung cancer we identified (Old age (especially >60 years), male sex, smoking history, and presence of the CAOD) would contribute in some way to the pathophysiology of AAA. Male sex is a well-known major predisposing factor (4–6 times more prevalent in men) in AAA development, consistent with our results. Previous studies found that endogenous sex hormone signaling pathways contributed to sex differences in AAA; androgens stimulate key pathological processes in AAA, while estrogen inhibits these processes [16]. Moreover, CAOD and AAA are closely related; the prevalence of CAOD in AAA is significantly higher than that in the general population and vice versa [17]. It is unclear whether the strong association is simply due to shared risk factors or if there are other causes beyond that [18]. Smoking is a predominant risk factor for not only lung cancer but also AAA. Smoking is known to contribute to AAA’s growth rate of up to 0.4 mm per year [19]. Moreover, there is a dose-dependent relationship between smoking and AAA; smoking duration and total lifetime smoking exposure both directly correlate with an increased risk of AAA [20].

In our study, AAA prevalence in smokers was 9-fold higher than that in non-smokers, which is consistent with previous reports that smoking is an important risk factor for developing AAA [21,22] but which is much higher than previously reported results (2–5-fold) [23,24]. This could be related to the ethnicities of the study population. It is well known that AAA is most frequent in the Caucasian population, and the Asian population is the least frequently affected by AAA according to available data on the frequency of AAA in different races [25]. Since our database comprises Korean individuals, we cannot directly compare the AAA incidence rates between Asians and Caucasians. Additionally, it is already known that not only ethnicity but also smoking history and smoking amounts (pack-years) affect the incidence and prevalence of AAA [23]. A previous retrospective study about AAA prevalence in lung cancer patients in the US, which had a higher rate of smokers (about 90%), showed that patients who smoked were more likely than nonsmokers to have AAA (11.9% [95% CI, 9.8–14.6] vs 2.2% [95% CI, 0.1–8.1]; P = 0.0021) [26]. The absolute incidence rate of AAA in smokers is higher than our study results. The reason for the higher proportion of AAA incidence in smokers compared to nonsmokers, as observed in previous studies involving Caucasians, might be because, in our study group, patients with lung cancer were highly likely to be heavy smokers. Because the number of smokers in our study is high (about 50%), the prevalence of AAA might be influenced by multiple factors, including ethnicity, smoking history, and the amount of smoking. And, the trend that the incidence rate of AAA is lower in Asians is consistent with previous studies in our study even in smokers. Moreover, smoking amount showed a dose-dependent relationship with the prevalence rate of AAA in patients with lung cancer (OR, 1.02; 95% CI, 1.01–1.03; P<0.001; Table 4), similar to previous study results [23]. As older age is an important risk factor for AAA development, this difference may have contributed to the higher prevalence of AAA in the lung cancer group. However, multivariable logistic regression analysis revealed that the prevalence of AAA was 10-fold higher in the lung cancer group than in the cancer-free control group, even after adjusting for age, sex, smoking history, and other AAA risk factors (Table 4); this suggests a possibility that lung cancer is a potential independent risk factor for AAA development.

Although the prevalence of AAA in patients with lung cancer was significantly higher than that in our control group, it was lower than that previously reported (3.8% vs. 11.1%) [26]. Even among patients with lung cancer aged >65 years, the prevalence of AAA was 6%. This could be because AAA prevalence is lower in Asians than in Caucasians [27]. Another possible reason is that the study population of previous studies included higher proportions of patients with advanced lung cancer, squamous cell lung cancer, and small cell lung cancer, which are known to be associated with heavy smoking [28]. Whereas, almost half of our study population were never smokers. In the univariable logistic regression model, squamous cell lung cancer was related to a higher risk of AAA development than adenocarcinoma. This might be related to the fact that squamous cell lung cancer is more strongly related than adenocarcinoma to smoking [29].

Current guidelines recommend interventional treatment (surgical or endovascular repair) only when the AAA diameter exceeds 5.5 cm. For small AAAs (3.0–5.4 cm), regular monitoring with ultrasonography or CT regularly based on its diameter is recommended [30]. Former smokers who quit smoking for >25 years have a similar relative risk of developing AAA as that of never smokers [31]. Furthermore, quitting smoking also reduces the risk of AAA by about 30% every 10 years [23]. Thus, smoking cessation is important for lung cancer and AAA surveillance and reducing the growth rate of AAA.

Several studies have investigated medications for AAA aimed at reducing aortic inflammation and proteolysis and supporting vascular smooth muscle cell recovery. However, there is no strong scientific evidence that supports pharmacological treatment to reduce AAA growth in humans [32,33]; the benefit of pharmacologic therapies, such as statins [34], antihypertensive drugs (beta blockers and angiotensin-converting enzyme inhibitors) [35,36], metformin [37], and antibiotics (roxithromycin and doxycycline) [38,39] in preventing rupture in small AAAs is controversial. However, there is some evidence that high blood pressure increases the risk of developing AAA [40]. Therefore, strict control of blood pressure in patients with lung cancer with AAA might be helpful as a preventive strategy for AAA complications.

Based on the proven cost-effective benefit of population-based AAA screening programs in high-risk groups [2], the US Preventive Services Task Force (USPSTF) recommends screening with ultrasonography for patients at high risk of AAA (men 65 to 75 years of age with a history of smoking). A previous study found that AAA prevalence is higher in patients with lung cancer [26]. In our study, the prevalence of AAA was also significantly higher in the resectable lung cancer group, indicating that patients with lung cancer are at high risk of AAA. Unlike previous studies, we evaluated the prevalence of AAA in patients with early lung cancer. Because patients with early lung cancer have a much longer life expectancy than that of patients with advanced-stage lung cancer, timely management of AAA in early lung cancer would have a greater advantage in preventing acute emergency and subsequent deaths from AAA rupture than in advanced-stage lung cancer. However, in our real-world database, only 6 out of 39 patients with lung cancer with AAA were diagnosed with AAA and managed by clinicians. The majority of AAAs in patients with lung cancer (84.6%; 33/39) were ignored without risk management to prevent AAA rupture.

There are several limitations of this study. First, there were limited information on several causal risk factors such as family history of vascular disease and lung cancer, stage of malignancy, or extent of the AAA because our database was retrospectively analyzed. Genetic factors and family history are well-known risk factors for both lung cancer [41] and AAA [42]. Therefore, a family history of lung cancer or AAA may be an important risk factor for AAA in patients with lung cancer. Second, the prevalence of AAA in the cancer-free group was lower than the general prevalence of AAA in Asian populations [27]. That might be related to the characteristics of our study population; the cancer-free control group was younger (mean age 54.4) than 65 years, which is the cut-off age for AAA surveillance [43]. In addition, those who voluntarily undergo regular health check-ups are likely to have a healthier lifestyle, including smoking cessation. Third, there is selection bias due to initially reviewing operable lung cancer patients and the limitation of retrospective analysis. However, it is an acceptable choice considering that surveillance is meaningful when its benefits outweigh the costs when applied to the general population. And, It is not clearly concluded whether it is a simple co-occurrence or if there is an association between AAA and lung cancer. Further studies are needed to determine the reason for the high prevalence of AAA in lung cancer patients.

It is unclear whether AAA surveillance is beneficial for patients with advanced lung cancer whose life expectancy is short. However, we expect that early detection and active monitoring of AAA would be beneficial for long-term survival in patients with early lung cancer because AAA rupture can be fatal, and the prognosis of early lung cancer is good.

In future studies, there is a need to evaluate the cost-effectiveness of the benefits of AAA surveillance in patients with lung cancer. In addition, further studies on medications to reduce aortic inflammation and proteolysis and enhance vascular smooth muscle cell recovery to reduce the complications of AAA are required.

## Conclusion

Our study found a meaningful relationship between lung cancer and AAA, indicating that early-stage lung cancer patients are at high risk for AAA and may benefit from AAA surveillance. Therefore, we suggest that early-stage lung cancer patients, especially those with a history of smoking or over the age of 60, be considered for regular AAA surveillance.

## Supporting information

S1 Table Baseline characteristics of AAA in the lung cancer group (LC) and cancer-free control group (CONT). (DOCX)

S1 Data (XLSX)

## Abbreviations

AAA: abdominal aortic aneurysm
BMI: body max index
HTN: hypertension
DM: diabetes mellitus
COPD: chronic obstructive pulmonary disease
CAOD: coronary artery obstructive disease
PAOD: peripheral arterial occlusive disease
NSCLC: non-small cell lung cancer
SCC: squamous cell carcinoma
CKD: chronic kidney disease
CI: confidence interval
OR: odds ratio
PY: pack years; y/n, yes/no
APCT: abdominal-pelvic computed tomography
PET: positron emission tomography
