# Supplementary material for: Cross-sectional study of lung cancer patients as a potential high-risk factor for abdominal aortic aneurysm
Source: PLoS One. 2025 Jan 6;20(1):e0315898. doi: 10.1371/journal.pone.0315898 (PMC11703101; doi:10.1371/journal.pone.0315898)
Supplement: S1 Table — (DOCX) [file pone.0315898.s001.docx]

S1 Table. Baseline characteristics of AAA in the lung cancer group (LC) and cancer-free control group (CONT)

| **Group** | **No** | **Sex** | **Age** | **BMI** | **smoking status (PY)** | **Lung cancer pDx†** | **AAA Tx** | **Size**  **(cm)** | **AAA type** | **HTN** | **DM** | **dyslipidemia** | **COPD** | **CAOD** | **PAOD** |
| --- | --- | --- | --- | --- | --- | --- | --- | --- | --- | --- | --- | --- | --- | --- | --- |
| **LC** | **1** | M | 65 | 26.5 | Current (45) | Adenoca | 2 | 3.1 | Juxtarenal | No | No | No | No | No | No |
| **LC** | **2** | M | 74 | 22.3 | Former (50) | SCCa | 2 | 3.1 | Infrarenal | Yes | Yes | No | Yes | Yes | No |
| **LC** | **3** | M | 72 | 21.9 | Never | Adenoca | 2 | 3.1 | Suprarenal | Yes | Yes | No | No | No | No |
| **LC** | **4** | M | 75 | 28.8 | Former (94) | Adenoca | 1 | 5.1 | Infrarenal | Yes | No | Yes | No | No | No |
| **LC** | **5** | M | 76 | 25.5 | Former (50) | Adenoca | 0 | 4.6 | Infrarenal | Yes | Yes | Yes | Yes | Yes | No |
| **LC** | **6** | M | 66 | 25.8 | Current (50) | Adenoca | 2 | 3.4 | Infrarenal | No | No | No | Yes | No | No |
| **LC** | **7** | M | 77 | 21.8 | Former (40) | SCCa | 2 | 3.2 | Pararenal | Yes | No | No | Yes | No | No |
| **LC** | **8** | M | 77 | 23.5 | Never | Adenoca | 2 | 3 | Infrarenal | Yes | No | Yes | No | Yes | No |
| **LC** | **9** | M | 77 | 26.4 | Former (25) | SCCa | 2 | 3.1 | Suprarenal | Yes | No | No | No | No | No |
| **LC** | **10** | M | 79 | 23.6 | Former (40) | Adenoca | 1 | 5.3 | Infrarenal | Yes | Yes | Yes | No | Yes | No |
| **LC** | **11** | M | 68 | 25.9 | Current (48) | Adenoca | 2 | 3.1 | Infrarenal | Yes | Yes | Yes | Yes | Yes | No |
| **LC** | **12** | M | 71 | 31.3 | Former (21) | Adenoca | 2 | 3 | Suprarenal | Yes | No | No | No | No | No |
| **LC** | **13** | M | 63 | 25.6 | Current (35) | Adenoca | 2 | 3.5 | Suprarenal | No | No | No | No | No | No |
| **LC** | **14** | M | 60 | 24.9 | Former (16) | Adenoca | 2 | 3 | Suprarenal | No | No | No | No | No | No |
| **LC** | **15** | M | 78 | 20.4 | Current (76) | SCCa | 2 | 4.3 | Infrarenal | Yes | Yes | Yes | Yes | Yes | Yes |
| **LC** | **16** | M | 73 | 20.2 | Former (47) | Adenoca | 2 | 3.2 | Infrarenal | No | No | No | No | No | No |
| **LC** | **17** | M | 62 | 26.9 | Former (20) | SCCa | 2 | 3.2 | Suprarenal | Yes | No | Yes | No | Yes | No |
| **LC** | **18** | M | 59 | 27.6 | Former (34) | Adenoca | 2 | 3.1 | Infrarenal | Yes | No | No | No | No | No |
| **LC** | **19** | M | 71 | 21.3 | Current (51) | SCCa | 2 | 3.1 | Suprarenal | Yes | No | No | Yes | No | No |
| **LC** | **20** | M | 79 | 22.9 | Former (48) | SCCa | 0 | 3.5 | Infrarenal | Yes | No | No | Yes | Yes | No |
| **LC** | **21** | M | 79 | 23.3 | Current (14) | Adenoca | 2 | 3.1 | Suprarenal | No | No | No | Yes | No | No |
| **LC** | **22** | F | 78 | 23.9 | Never | Adenoca | 0 | 3.1 | Suprarenal | Yes | No | Yes | No | No | No |
| **LC** | **23** | M | 67 | 21.1 | Former (30) | Adenoca | 0 | 3 | Infrarenal | No | Yes | No | Yes | Yes | No |
| **LC** | **24** | M | 60 | 29.5 | Current (40) | Adenoca | 0 | 4.5 | Infrarenal | Yes | No | Yes | Yes | Yes | No |
| **LC** | **25** | M | 81 | 24 | Current (50) | SCCa | 2 | 3.3 | Pararenal | Yes | No | No | Yes | Yes | No |
| **LC** | **26** | M | 69 | 19.5 | Former (46) | SCCa | 0 | 3.3 | Infrarenal | Yes | No | Yes | Yes | Yes | No |
| **LC** | **27** | M | 76 | 20.4 | Current (56) | SCCa | 2 | 3.1 | Infrarenal | Yes | No | No | No | Yes | No |
| **LC** | **28** | M | 78 | 22.3 | Never | Others | 2 | 3 | Juxtarenal | Yes | No | No | No | No | No |
| **LC** | **29** | M | 73 | 25.6 | Former (40) | Adenoca | 2 | 4.4 | Infrarenal | Yes | Yes | Yes | Yes | Yes | No |
| **LC** | **30** | M | 64 | 24.2 | Current (45) | SCCa | 2 | 3 | Suprarenal | Yes | Yes | No | Yes | No | No |
| **LC** | **31** | M | 62 | 25 | Current (49) | Adenoca | 2 | 3 | Infrarenal | No | No | No | No | No | No |
| **LC** | **32** | M | 70 | 19.1 | Current (80) | SCCa | 2 | 3.3 | Infrarenal | Yes | No | Yes | Yes | No | No |
| **LC** | **33** | M | 65 | 19.9 | Current (40) | Adenoca | 2 | 3.6 | Infrarenal | Yes | Yes | Yes | No | No | No |
| **LC** | **34** | M | 64 | 25.6 | Former (40) | Adenoca | 2 | 3 | Infrarenal | Yes | Yes | Yes | Yes | No | No |
| **LC** | **35** | M | 66 | 25.3 | Former (44) | SCCa | 2 | 3.2 | Infrarenal | Yes | No | Yes | Yes | No | No |
| **LC** | **36** | M | 67 | 23.9 | Former (43) | SCCa | 2 | 3 | Pararenal | Yes | No | No | Yes | No | No |
| **LC** | **37** | M | 80 | 20.8 | Former (10) | Adenoca | 2 | 3.1 | Suprarenal | No | No | No | No | No | No |
| **LC** | **38** | M | 66 | 21.8 | Former (45) | Adenoca | 2 | 3 | Infrarenal | Yes | No | No | No | No | No |
| **LC** | **39** | M | 80 | 24.2 | Former (12) | Adenoca | 2 | 3.6 | Infrarenal | Yes | Yes | Yes | Yes | No | No |
| **LC** | **40** | M | 82 | 22.3 | Former (30) | Adenoca | 2 | 3 | Infrarenal | Yes | No | No | No | No | No |
| **LC** | **41** | M | 71 | 23.7 | Current (75) | Others | 2 | 4.6 | Infrarenal | No | No | No | No | No | No |
| **Cont** | **1** | M | 48 | 22.9 | Current (14.5) | - | 2 | 5.3 | Infrarenal | No | No | No | No | No | No |
| **Cont** | **2** | M | 67 | 22.5 | Former (49) | - | 2 | 4.5 | Suprarenal | Yes | No | No | No | No | No |
| **Cont** | **3** | M | 59 | 25.3 | Former (10) | - | 1 | - | Suprarenal | Yes | No | No | No | No | No |
| **Cont** | **4** | M | 58 | 27.4 | Former (39) | - | 2 | 3.0 | Infrarenal | Yes | No | Yes | No | No | No |
| **Cont** | **5** | M | 69 | 23.9 | Current (50) | - | 2 | 3.0 | Infrarenal | Yes | No | No | No | No | No |
| **Cont** | **6** | M | 59 | 23.8 | Former (20) | - | 2 | 3.0 | Infrarenal | Yes | No | No | No | No | No |
|  | AAA : abdominal aortic aneurysm, LC: lung cancer group, CONT: control group, PY : pack-year, HTN : hypertension, DM : diabetes mellitus, COPD : chronic obstructive pulmonary disease, CAOD : coronary artery obstructive disease, PAOD : peripheral artery occlusive disease  † Lung cancer pDx : lung cancer pathologic diagnosis  SCCa : squamous cell carcinoma  Other lung cancer: large cell, adenosquamous, carcinoid, sarcomatoid carcinoma, large cell neuroendocrine carcinoma, small cell-large cell carcinoma  AAA Tx : 0 : regular follow up, 1: operation/intervention, 2: no more follow up  AAA Type : AAA classification according to the location of an aneurysm | | | | | | | | | | | | | | |
